# Is Pyroglutamic Acid a Prognostic Factor Among Patients with Suspected Infection? A Prospective Cohort Study

**DOI:** 10.1038/s41598-020-66941-7

**Published:** 2020-06-23

**Authors:** Itai Gueta, Yarden Perach Ovadia, Noa Markovits, Yehoshua N Schacham, Avi Epsztein, Ronen Loebstein

**Affiliations:** 1 The institute of Clinical Pharmacology and Toxicology, Sheba Medical Center, Tel Hashomer, Israel; 2 Department of Medicine A, Sheba Medical Center, Tel Hashomer, Israel; 3 Department of Emergency Medicine, Sheba Medical Center, Tel Hashomer, Israel; 4 Sackler School of Medicine, Tel Aviv University, Tel Aviv, Israel

**Keywords:** Predictive markers, Prognostic markers, Infectious diseases

## Abstract

Pyroglutamic acid (PGA) is a compound that accumulates during oxidative stress and hence, elevated levels may be associated with poor prognosis in patients with infection or sepsis. To examine this hypothesis, patients presenting with acute infection were recruited in the emergency department and prospectively followed for 30 days. Sport urine samples were quantified for PGA. Outcomes were mortality and composite outcome of death or organ failure. Thirty two (32%) patients had qSOFA≥2. Median urine PGA was 22.9 (IQR 17.64, 33.53) µmol/mmol creatinine. Four patients demonstrated PGA values ≥ 63 µmol/mmol creatinine. Univariate analysis showed that PGA concentration ≥ 75^th^ percentile (i.e. 33.53 µmol/mmol creatinine) was associated with higher rates of in-hospital mortality (p = 0.041) with similar trend for PGA ≥ 63 µmol/mmol creatinine (p = 0.04). However, multivariate analysis showed that PGA was not associated with worse outcomes, whereas heart rate was associated with both composite outcomes (HR 1.0, p = 0.008 and HR 1.02, p = 0.001 for composite outcome with 30 days and in-hospital mortality, respectively). Among low risk patients, high PGA levels were consistently associated with worse outcomes. In conclusion, urine PGA concentration was not associated with worse outcomes among septic patients. Nevertheless, future studies should evaluate this association in larger cohorts.

## Introduction

Sepsis is a leading cause of in-hospital mortality with varying rates between 20% and 30%^1,2^. Over the last decades an increasing incidence has been documented, probably due to the increasing use of immunosuppressive agents, occurrence of resistant microbes and the aging population^1,3^. Sepsis induced tissue ischemia and organ injury is partly due to micro- and macrocirculatory derangement^4,5^. The latter is evaluated and monitored by hemodynamic and laboratory parameters such as blood pressure, urine output and serum creatinine, which among others have been included in several scores aiming to identify patients with sepsis and high mortality risk. These include logistic organ dysfunction system (LODS), sequential organ failure assessment (SOFA) and quick SOFA (qSOFA). However, their accuracy in predicting mortality varies^6–8^.

In recent years much attention has been given to infection and sepsis-associated microcirculatory injury and related markers for oxidative stress, free radical accumulation (reactive oxygen species), glutathione depletion and impaired mitochondrial activity^9^. However, only few markers have been shown to be associated with worse clinical outcomes^4,9–11^. Accordingly, various studies have examined the effect of antioxidant therapy (e.g. vitamin C, N-acetylcysteine (NAC), selenium) on survival of septic patients, with conflicting results^4,12,13^.

Pyroglutamic acid (PGA) is a compound in the gamma-glutamyl cycle. Its accumulation, resulting in high anion gap (AG) metabolic acidosis, was demonstrated in oxidative stress- induced glutathione depletion states, such as chronic toxicity of supratherapeutic doses of paracetamol^14,15^. In light of these observations we hypothesized that oxidative stress due to infection and sepsis, along with its associated glutathione depletion, may be also associated with elevated PGA levels, which may further accumulate due to decreased renal perfusion and PGA clearance^16^. The objective of the current study was to examine whether PGA concentration is associated with organ failure and mortality among patients with suspected infection or sepsis.

## Material and Methods

### Study Design and Cohort definition

We conducted a prospective observational study between November 2018 and April 2019 at the Emergency Department (ED) of a large tertiary hospital in Israel. Subjects ≥ 18 years old arriving to the ED with a clinical presentation suggestive of infection defined as: (1) The presence of fever ≥ 38.0 Celsius in the preceding 24 hours and (2) Clinical (e.g. urinary or respiratory complaints), laboratory (e.g. neutrophil left deviation, pathologic urine analysis) or radiographic evidence for infection. Oncologic patients and patients with neutropenic fever were excluded. The study was approved by the institutional Review Board (Helsinki committee of Sheba Medical Center, approval code: 5291-18-SMC) and an informed consent was obtained from all participants or their legal guardians. All methods were carried out in accordance with relevant guidelines and regulations.

### Data extraction

Data collected for each patient included demographic characteristics, comorbidities, chronic medications, number of hospitalizations in the month prior to index hospitalization and main diagnosis. ED data included hemodynamic parameters (blood pressure, pulse, respiratory rate), laboratory values (complete blood count, blood chemistry panel, blood gases, calculated anion gap, lactate and urine ketones) and time until first antibiotic therapy. For each patient, qSOFA score was calculated. Follow up was defined as the time from admission to death or 30 days. Cases in which patients were discharge before 30 days, the investigator (IG or YPO) contacted them or their family member via phone call.

### Definitions and endpoints

#### Pyroglutamic acid levels

Spot urine samples taken shortly after arrival to ED were tested for PGA concentration using gas chromatograph mass spectrometry. The analyses were performed on a Hewlett-Packard (PaloAlto, CA) HP5890A gas chromatograph coupled to an HP5970B mass-selective detector and an HP59940A ChemStation. Quantification of the acids was based on the specific ion masses^17^. Increased PGA concentrations were defined as:

1. PGA ≥ 63 µmol/mmol creatinine, based on the values described in the general population^18^.

2. PGA ≥ 75th percentile of the sample population.

#### Outcomes

The primary outcome was 30-days or in-hospital mortality. Secondary outcomes were defined as composite outcome, analyzed separately for the combination of 30 days mortality or in-hospital mortality together with at least one organ failure defined as:

1. Kidney failure – defined as creatinine rise ≥ 150% of baseline.

2. Invasive ventilation.

3. Intravenous amines treatment.

Additionally, an adjusted qSOFA score was calculated by adding 1 point for patients with low qSOFA score (i.e. ≤1) and elevated PGA levels as defined above.

### Data analysis

Comparisons between groups were conducted using paired t-tests or Mann-Whitney U test for parametric and non-parametric analysis, respectively. Categorical variables were compared using Chi-square or Fisher’s exact test. Kaplan-Meier analysis was employed to examine the association between PGA levels and mortality. Composite outcomes were analyzed using logistic regression models. The latter included confounders that were demonstrated to be significant in the univariate analysis. Additional sub-analysis was conducted for patients with high and low mortality risk (qSOFA above and below 2, respectively). Finally, qSOFA and adjusted qSOFA area under the Receiver Operating Characteristic (AUROC) curve and its associated terms were calculated and their 95% CI compared. Missing values were omitted from the analysis. All analyses were 2-tailed and P ≤ 0.5 was considered significant. All statistical analyses were performed by using SPSS software (version 25 IBM, SPSS Inc).

## Results

A total of 103 patients were recruited, of whom 3 were excluded due to technical problems in PGA determination. The final cohort included 43 females and 57 males, with a median age of 79.5 (IQR 67, 87) years. Rates of hypertension, ischemic heart disease, diabetes mellitus and dyslipidemia were 62%, 21%, 37% and 42%, respectively. Mean heart rate and blood pressure on arrival to ED were 99 (±19.8) bpm and 130(±31)/72(±17.8) mmHg, respectively. 15% of patients demonstrated systolic blood pressure ≤100 mmHg and 9% had mean arterial pressure ≤ 65 mmHg. Median pH was 7.39 (7.35, 7.41) and the median anion gap was 10.3 mEq/L (8.73, 12.45). Anion gap ≥ 11 was demonstrated among 32/80 (40%) patients, of whom 22/80 (27.6%) had an AG ≥ 12 and 5/80 (6.3%) ≥ 16. Thirty two (32%) patients had qSOFA ≥ 2, of them 7/32 and 1/32 with score of 3 and 4, respectively. One patient consumed therapeutic dose of paracetamol in the preceding 48 hours and none reported chronic paracetamol use. Baseline characteristics and laboratory values are presented in Table 1 and Table 2.

**Table 1:** Baseline characteristics.

	PGA < 33.5 µmol/mmol creatinine	PGA ≥ 33.5 µmol/mmol creatinine	Total	P value
N	75 (75%)	25 (25%)	100	
Gender, Female	28 (37.3%)	15 (60%)	43(43%)	0.047
Age (IQR)	78 (62, 92)	84 (71, 92)	79.5 (67,87)	0.11
BMI ≤ 18 kg/m^2^	2/24 (8.3%)	0/3 (0%)	2 (2%)	1.0
Hypertension	45 (60%)	17 (68%)	62 (62%)	0.475
Ischemic heart disease	17 (22.7%)	4 (16%)	21	0.478
Congestive heart failure	12 (16%)	3 (12%)	15	0.755
Diabetes mellitus	24 (32%)	13 (52%)	37	0.073
S/P Stroke	24 (32%)	10 (40%)	34	0.465
Dyslipidemia	29 (38.7%)	13 (52%)	42	0.242
Atrial fibrillation	12 (16%)	7 (28%)	19	0.239
Chronic kidney disease	9 (12%)	5 (20%)	14	0.33
α Blockers	14 (18.7%)	3 (12%)	17 (17%)	0.55
β Blockers	22 (29.3%)	7 (28%)	29	0.899
Calcium channel blockers	16 (21.3%)	4 (16%)	20	0.564
ACEI/ARBs	31 (41.3%)	8 (32%)	39	0.407
Diuretics	13 (17.3%)	8 (32%)	21	0.119
Antipsychotics	6 (8%)	6 (24%)	12	0.053
Platelet aggregation inhibitors	33 (44%)	9 (36%)	42	0.841
Anticoagulants	13 (65%)	7 (28%)	20	0.248
Statins	21 (28%)	2 (8%)	23	0.04

**Table 2:** Laboratory and hemodynamic values at arrival to emergency department.

Variable (Normal values)	PGA < 33.5 µmol/mmol creatinine	PGA ≥ 33.5 µmol/mmol creatinine	Total	P value
Hemoglobin (12–16), g/dl	12.4 (10.89, 13.8)	12.04 (9.67, 13.59)	12.37 (10.6, 13.65)	0.197
WBC (4–10.8), x103/microL	11.0 (8.22, 15.89)	11.8 (8.53, 14.12)	11.88 (8.33, 15.63)	0.63
Creatinine (0.51–0.95), mg/dl	0.98 (0.7, 1.56)	0.95 (0.64, 1.43)	0.97 (0.70, 1.53)	0.75
Urea (15–45), mg/dl	47 (30, 79)	69 (41.5, 104)	48 (33, 83.75)	0.053
Na (136–148), mEq/l	137 (134, 139)	138 (133, 144.5)	137 (134, 140)	0.136
K (3.5–5.2), mEq/l	4.2 (3.9, 4.7)	4.5 (3.95, 4.9)	4.2 (3.925, 4.775)	0.169
Cl (8.1–10.4), mEq/l	101 (98, 105)	102 (96.5, 108)	101 (98, 106)	0.327
ALT (7–40), IU/l	19 (12, 36.75)	24.5 (17.25, 52.75)	19 (13.28, 38)	0.185
Albumin (3.6–5.5), g/dl	3.6 (3.3, 3.9)	3.3 (2.6, 3.55)	3.5 (3.2, 3.8)	≤ 0.001
CRP (0–5), mg/l	91 (48, 164)	127.5 (37.09, 166.25)	94.3(45.34, 164)	0.457
Lactate (6–18), mg/dl	50 (14, 30.5)	21 (16, 36.43)	21(15, 31.5)	0.489
pH (7.31–7.42)	7.38 (7.34, 7.40)	7.40 (7.37, 7.45)	7.38 (7.34, 7.40)	0.039
HCO3 (22–27), mmol/l	25.85 (22.25, 27.7)	26.25 (23.58, 27.83)	26.05 (22.75, 27.7)	0.785
Anion gap, (8–12), mEg/l	9.75 (8.6, 11.93)	11.15 (9.78, 13.9)	10.35 (8.72, 12.45)	0.106
Ketones	11/39 (28.2%)	9/14 (64.3%)	20/53 (37.7%)	0.017
pCO2 (38–52), mmHg	43.25 (38.53, 48.55)	39.55 (35.8, 48.58)	42.75 (37.22, 48.55)^c^	0.255
SBP ≤ 100 mmHg	23 (16%)	3 (12%)	26	0.755
qSOFA ≥ 2	22 (29.3%)	10 (40%)	32	0.322

### PGA concentration

Median urine PGA was 22.9 (IQR 17.64, 33.53) µmol/mmol creatinine. Only 4% of the patients demonstrated above normal PGA values (defined as ≥ 63 µmol/mmol creatinine) ranging between 63.65 to 103.4 µmol/mmol creatinine. This group was characterized by lower hemoglobin level, albumin concentration and lower SBP on arrival (Available in supplementary information, Table S1). Univariate analysis comparing PGA concentrations below and above 75^th^ percentile (i.e. 33.53 µmol/mmol creatinine) demonstrated among the latter higher rates of females [15/25 (60%) vs 28/75 (37.3%), p = 0.047] and lower prevalence of statin use [2/25 (8%) vs 21/75 (28%), p = 0.040]. These patients were also characterized by lower albumin levels (3.3 g/dl vs 3.6 g/dl, p < 0.001), higher rates of urine ketones [11/39 (64.3%) vs 9/14 (28.2%), p = 0.017] and also higher, though not clinically significant, pH (7.4 vs 7.38, p = 0.039). Anion gap did not defer between groups (p > 0.05).

### Mortality

During 30 days follow up a total of 16 patients died with a mean time to death from hospitalization of 10.6 (±8) days, of whom 9/16 (56.3%) died during their hospital stay after a mean of 5.8 (±6.3) days. The latter group demonstrated higher rates of patients with SBP ≤ 100 mmHg and MAP ≤ 65 mmHg [4/9 (44.4%) vs 11/91 (12.1%), p = 0.027; 3/9 (33.3%) vs. 6/91 (6.6%), p = 0.033, respectively] and was also higher rates of chronic anemia per diagnosis [3/9 (33.3%) vs 7/91 (7.7%), p = 0.044] and low hemoglobin concentration (median 10.2 vs 12.45, p = 0.048). None of the patients suffered from bleeding. Additionally, these patients demonstrated higher median values of creatinine and urea, suggesting pre-renal kidney injury (1.72 vs 0.95 mg/dl, p = 0.06 and 133 vs 47 mg/dl, p = 0.04, respectively). PGA concentration ≥ 75^th^ percentile was associated with higher rates of in-hospital mortality [5/9 (55.6%) vs 20/91 (22%), p = 0.041) with similar trend for PGA ≥ 63 µmol/mmol creatinine (p = 0.04, supplementary information, Table S1). However, Kaplan-Meier analysis did not demonstrate significant association between PGA level (above and below 33.5 µmol/mmol creatinine) and 30 days or in-hospital mortality (p = 0.743 and p = 0.319, respectively).

### Composite outcome

Twenty six patients (26%) reached the composite outcome of 30 days mortality, acute renal failure, mechanical ventilation and amine use. Univariate analysis showed that these patients had higher rates of hospitalization due to infection during the preceding month [3/74 (4.1%) vs. 5/26 (19.2%), p = 0.027]. These patients were also characterized by lower median values of hemoglobin and albumin (11.32 vs 12.53 g/dl, p = 0.044 and 3.35 g/dl vs 3.6 g/dl, p = 0.007, respectively), and higher concentrations of urea and lactate (78.5 vs 45 mg/dl, p < 0.001 and 25 vs 19.5 mg/dl, p = 0.033, respectively). Interestingly, PGA ≥ 33.5 µmol/mmol creatinine was significantly associated with the composite outcomes [11/26 (42.3%) vs. 14/74 (18.4%), p = 0.018], with a trend that did not reach statistical significance among patients with PGA ≥ 63 µmol/mmol creatinine [3/26 (11.5%) vs. 1/74 (1.3%), p = 0.053].

Twenty (20%) patients reached the composite outcome that included in-hospital mortality. Univariate analysis showed similar trends as describe above for albumin, urea and lactate as well as higher rates of patients with PGA ≥ 75^th^ percentile [9/20 (45%) vs. 16/80 (20%), p = 0.021], (Available in supplementary information, Table S2). Logistic regression demonstrated that PGA ≥ 33.5 µmol/mmol creatinine was significantly associated with both composite outcome (composite outcome with 30 days mortality: HR 3.14, 95% CI 1.19–8.3, p = 0.021; composite outcome with in-hospital mortality: HR 3.27, 95%CI 116–9.24, p = 0.025). However, multivariate analysis adjusted for potential confounders showed that only albumin, urea and heart rate were significantly associated with either outcome, with no effect of PGA level (Table 3).

**Table 3:** Multivariate analysis for composite outcomes.

	β	95% CI	P value
**Composite outcome with 30 days mortality**			
Albumin	0.16	0.05–0.44	0.001
Heart rate	1.04	1.01–1.07	0.008
Constant	3.83		0.510
**Composite outcome with in-hospital mortality**			
Urea	1.05	1.02–1.09	0.001
Heart rate	1.02	1.01–1.04	0.001
Constant	0		<0.001

### Sub-analysis of patients with qSOFA ≥ 2 and qSOFA ≥ 1

Sub-analysis including only patients with qSOFA ≥ 2 (n = 32) demonstrated no differences in the rates of in-hospital mortality, 30 days mortality or composite outcomes between either PGA groups for either cutoffs. Interestingly, when analyzing separately only patients with low mortality risk (i.e. qSOFA ≤ 1, n = 68), significantly higher rates of in-hospital mortality and 30 days mortality were observed among those with PGA ≥ 75^th^ percentile (13.3% vs. 0%, p = 0.05 and 26.7% vs. 5.7%, p = 0.04, respectively). This trend was also observed for the composite outcome including in-hospital mortality (p = 0.06) with significantly higher rates for composite outcome with 30 days mortality (46.7% vs. 15.1%, p = 0.02). Unadjusted logistic regression showed significant association between the latter outcome and hemoglobin, urea and PGA ≥ 33.5 µmol/mol creatinine. Of these, only urea and PGA level kept their significant contribution in adjusted analysis (HR 1.02, 95%CI 1.0–1.04, p = 0.03 and HR 4.75, 95%CI 1.09–20.66, p = 0.04, respectively). Data available in supplementary information, Table S3 and S4a-c.

### qSOFA, Adjusted qSOFA and AUROC curve

Seven patients (21.9%) of those with qSOFA ≥ 2 died during their hospitalization. qSOFA demonstrated sensitivity of 77.8% (7/9) and specificity of 72.5% (66/91). The PPV and NPV were calculated to be 21.9% (7/32) and 97.1% (66/68), respectively. The adjusted qSOFA showed higher sensitivity (100%, 9/9) and lower specificity (62.6%, 57/91), PPV of 20.9% (9/43) and NPV of 100% (57/57). However, comparing AUROC curves demonstrated somewhat higher AUC but with comparable 95% CI (0.825, 95%CI 0.69–0.96 vs. 0.872, 95%CI 0.78–0.96).

## Discussion

The present study demonstrates that elevated PGA levels among patients with probable infection and high mortality risk has no major role in predicting worse outcomes. While unadjusted analysis demonstrated significant association between PGA levels, in-hospital mortality and composite outcomes, multivariate analysis revealed that cardiovascular state (i.e. heart rate and plasma albumin) are more important predictors for infection-induced complications. Interestingly, patients with elevated PGA levels were characterized by lower albumin and a trend towards higher urea concentrations. These may reflect their catabolic state and probably disease severity, which is further supported by higher rates of in-hospital mortality. However, rates of patients with qSOFA ≥ 2 did not differ between PGA groups. Additionally, no significant association was noted for composite outcome, either with or without mortality.

Normal values of PGA are commonly defined as ≤ 63 µmol/mmol creatinine. However, previous analytical studies among healthy adults reported upper limits as low as 32.6 µmol/mmol creatinine and 54 µmol/mmol creatinine^18^. Since only 4/100 patients in our cohort demonstrated elevated levels, we also evaluated a lower cutoff according to the 75^th^ percentile (i.e. 33.5 µmol/mmol creatinine). However, the latter did not demonstrated significant association with either outcome.

Our cohort reflected well qSOFA ≥ 2 sensitivity and specificity for in-hospital mortality, reported to range from 56 to 75% with a positive likelihood ratio of 1.6 and 2.3^6^. Interestingly, sub-analysis showed that among patients with acute infection that had not demonstrated organ dysregulation (i.e. qSOFA ≤ 1), higher PGA levels were significantly associated with mortality and worse outcomes, both in the univariate and multivariate analysis. This observation, concluded from the sub-analysis only, may reflect metabolic changes associated with oxidative stress that are yet to be observed clinically and may assist in identifying patients in higher mortality risk. Neverthless, as these observations are preliminary, we believe that its prognostic role cannot be definitively determined. In light of the latter, together with the significant association in the univariate analysis between PGA and in-hospital mortality, we further examined the impact of high PGA values on low qSOFA score and ROC curve terms. This demonstrated to increase the sensitivity to 100% and PLR to 2.7. However, AUROC curve demonstrated significant 95% CI overlap, and by that excluding the additive value of such an adjustment.

Previous studies have shown that increased plasma concentration of oxidative stress markers (e.g. microRNA-25, nitrotyrosine) among septic patients are associated with poor outcomes, further strengthened by evidence for greater antioxidant potential among survivors^9,19,20^. Another *in vitro* study suggested a pivotal role of glutathione depletion in sepsis generated ROS and endothelial cell damage, with reduction in the latter with NAC pretreatment^16^. Accordingly, several studies have attempted to enhance antioxidant capacity by NAC administration, but reported conflicting results. This may be explained by the fact that NAC was given to all patients regardless of objective evaluation of oxidative stress, and although oxidative stress markers have been already recognized, they are mostly impractical for clinical use. Hence, given sepsis induced glutathione depletion, and the observed PGA accumulation in the glutathione depletion states, we sought to study its association with sepsis complications, with potential future benefit from NAC administration. However, in these patients we found no such clinically significant association. This lack of association may be explained by several limitations in our study: first, patients in our cohort were heterogeneous with respect to severity, with only a few demonstrating hemodynamic compromise. This is reflected by the higher than expected pH, low rate of MAP ≤ 65 mmHg and qSOFA ≥ 2. However, when focusing on patients with *a priori* low mortality risk, PGA was consistently associated with poor outcomes. This observation, though part of a sub-analysis only, may suggest a prognostic role. Additionally, while our study included a single spot urine sample for PGA, a previous study reported high within-individual PGA variability among healthy individuals, also affected by previous weeks of metabolic stress^21^. However, rates of previous hospitalization due to infection did not differ between groups and hence could partly account for this potential bias. Data on BMI and malnutrition was available only in 27 patients and hence we could not fully adjust the results for this variable. Nevertheless, none of patients had signs of severe malnutrition. Cancer-related cachexia was not a factor as patients with malignant disease were excluded from the study. Hence, we believe that malnutrition did not significantly biased out results. Lastly, as data about PGA concentrations among septic patient is unavailable, our sample could only be defined by the period during which patients were recruited; this may have underpowered our results in order to detect significant association between PGA and worse outcomes. However, in order to overcome this problem several analyses were conducted for different PGA cutoffs, with no significant change on the final result. Of note is the fact that current methods to measure pyroglutamic acid concentration is commonly conducted by GC/MS and cannot be routinely, and quickly, used for evaluation in the emergency room setting. If future studies will also demonstrate the prognostic value of high PGA concentration in patients with acute infection, easy and fast methods for PGA quantification must be evaluated.

In conclusion, our study demonstrates that urine pyroglutamic acid concentration is not associated with worse outcomes among patients with suspected infection and high mortality risk. Nevertheless, as our sample might have been too small, and in the light of the finding among low risk patients, future studies should consider examining this association among larger cohorts.

## Supplementary information

Supplementary information.
