# Supplementary material for: Is Pyroglutamic Acid a Prognostic Factor Among Patients with Suspected Infection? A Prospective Cohort Study
Source: Sci Rep. 2020 Jun 23;10:10128. doi: 10.1038/s41598-020-66941-7 (PMC7311518; doi:10.1038/s41598-020-66941-7)
Supplement: Supplementary file 1 — Supplementary information. [file 41598_2020_66941_MOESM1_ESM.pdf]

Supplementary Information

IS PYROGLUTAMIC ACID A PROGNOSTIC FACTOR AMONG PATIENTS WITH SUSPECTED INFECTION? A PROSPECTIVE COHORT STUDY

Itai Gueta MD[1,4], Yarden Perach Ovadia MD[2], Noa Markovits MD[1,4], Yehoshua N Schacham MD[1,4], Avi Epsztein MD[3], Ronen Loebstein MD[1,4]

[1]The institute for Clinical Pharmacology and Toxicology, [2]Department of Medicine A, [3]Department of Emergency Medicine, Sheba Medical Center, Tel Hashomer, Israel. [4]Sackler School of Medicine, Tel Aviv University, Tel Aviv, Israel.

Table S1. Univariate analysis according to PGA levels above and below 63 µmol/mmol creatinine

Variable	Normal value	PGA < 63 µmol/mmol creatinine	PGA ≥ 63 µmol/mmol creatinine	P value
**Hemoglobin**	12-16 g/dl	12.39 (10.72, 13.78)	9.46 (0.32, 11.8)	0.045
**Albumin**	3.6-5.5 g/dl	3.6 (3.2, 3.8)	2.3 (2.2, <2.3)	0.037
**Systolic blood pressure**		131 (107.3, 159)	99 (69.3, 112.25)	0.021
**In-hospital mortality**		7/96 (7.3%)	2/4 (50%)	0.040
**30 days mortality**		13/96 (13.5%)	3/4 (75%)	0.012

Table S2. Univariate analysis for composite outcome with in hospital mortality

Variable	Normal value	No (n=80)	Yes (n=20)	P value
**Heart rate**	60-90 bpm	96 (84, 106)	109.5 (93.5, 120)	0.008
**Lactate**	6-18 mg/dl	19 (14, 29)	26.85 (19.25, 45.8)	0.014
**Albumin**	3.6-5.5 g/dl	3.6 (3.3, 3.8)	3.4 (2.3, 3.7)	0.039
**Urea**	15-45 mg/dl	46 (29.25, 78.25)	74 (48.75, 142.75)	0.002
**PGA ≥ 33.5**		16 (20%)	9 (45%)	0.021
**PGA ≥ 63**		2 (2.5%)	2 (10%)	0.178
**Previous hospitalization due to infection**		4 (5%)	4 (20%)	0.049

Table S3. Subanalysis including only patients with qSOFA ≥ 2

Variable	PGA < 33.5 µmol/mmol creatinine	PGA ≥ 33.5 µmol/mmol creatinine	P value	PGA < 63 µmol/mmol creatinine	PGA ≥ 63 µmol/mmol creatinine	P value
**In-hospital mortality**	4/22 (18.2%)	3/10 (30%)	0.65	5/29 (17.2%)	2/3 (66.7%)	0.11
**30 days mortality**	6/22 (27.3%)	3/10 (30%)	1.0	7/29 (24.1%)	2/3 (66.7%)	0.18
**Composite outcome (with in-hospital mortality)**	5/22 (22.7%)	4/10 (40%)	0.41	7/29 (24.1%)	2/3 (66.7%)	0.18
**Composite outcome (with 30 days mortality)**	7/22 (31.8%)	4/10 (40%)	0.70	9/29 (31%)	2/3 (66.7%)	0.27

Tables S4a-4b. Sub-analysis including only patients with qSOFA ≤ 1

S4a. Univariate analysis comparing PGA groups

Variable	PGA < 33.5 µmol/mmol creatinine	PGA ≥ 33.5 µmol/mmol creatinine	P	PGA < 63 µmol/mmol creatinine	PGA ≥ 63 µmol/mmol creatinine	P
Epilepsy	1/53 (1.9%)	3/15 (20%)	0.03			
Ketones	6/24 (26.1%)	6/9 (66.7%)	0.05			
Albumin	3.6 (3.3, 3.83)	3.3 (2.86, 3.7)	0.03			
In-hospital mortality	0/53 (0%)	2/15 (13.3%)	0.05	2/67 (3%)	0/1 (0%)	1.0
30 days mortality	3/53 (5.7%)	4/15 (26.7%)	0.04	6/67 (9%)	1/1 (100%)	0.10
Composite outcome (with in-hospital mortality)	6/53 (11.3%)	5/15 (33.3%)	0.06	11/67 (16.7%)	0/1 (0%)	1.0
Composite outcome (with 30 days mortality)	8/53 (15.1%)	7/15 (46.7%)	0.02	14/67 (20.9%)	1/1 (100%)	0.22

S4b. Logistic regression for composite outcome with in-hospital mortality

	Unadjusted			Adjusted		
Variable	β	95% CI	P	β	95% CI	P
Recurrent hospitalization	10.31	1.49-71.53	0.02			NS
Hemoglobin	0.69	0.5-0.96	0.03	0.42	0.19-0.90	0.03
Albumin	0.17	0.04-0.83	0.03			NS
Urea	1.02	1.0-1.04	0.03			NS
Heart rate	1.05	1.01-1.09	0.03	1.13	1.03-1.24	0.01
PGA ≥ 33.5 µmol/mmol creatinine	3.92	1.0-15.4	0.05			NS

S4c. Logistic regression for composite outcome with 30 days mortality

	Unadjusted			Adjusted		
Variable	β	95% CI	P	β	95% CI	P
Hemoglobin	0.66	0.48-9.1	0.01			NS
Urea	1.03	1.01-1.04	0.01	1.02	1.00-1.04	0.03
PGA ≥ 33.5 µmol/mmol creatinine	4.92	1.39-17.4	0.01	4.75	1.09-20.66	0.04
